# Parasites influence the physiology and personality in a small mammal (*Ochotona curzoniae*)

**DOI:** 10.7717/peerj.20420

**Published:** 2025-11-28

**Authors:** Rong Wang, Suqin Wang, Jiapeng Qu

**Affiliations:** 1 Qinghai Province Key Laboratory of Animal Ecological Genomics, Xining, China; 2 School of Life Science, Qinghai Normal University, Xining, Qinghai, China; 3 Key Laboratory of Adaptation and Evolution of Plateau Biota, Northwest Institute of Plateau Biology, Chinese Academy of Sciences, Xining, Qinghai, China; 4 University of Chinese Academy of Sciences, Beijing, China

**Keywords:** Parasite, Plateau pika, Personality, Physiology, Qinghai-Tibetan plateau

## Abstract

**Background:**

Parasites are prevalent in animals and have coexisted with their hosts over long evolutionary periods. However, the link between individual behavioral variations and parasitic infections remains unclear. Plateau pika (*Ochotona curzoniae*) is a keystone species on the Qinghai-Tibetan Plateau that is commonly infected with *Eimeria* spp., an intestinal parasite.

**Methods:**

In this study, 30 adult pikas were assigned to three groups: infected with *Eimeria* spp. (PA+), administered normal saline (Ctrl), and treated with an anticoccidial drug (PA−). We examined changes in boldness, exploration, and docility, as well as variations in triiodothyronine (T3), thyroxine (T4), resting metabolic rate (RMR), and fecal cortisol (CORT) levels.

**Results:**

The PA+ group exhibited significantly higher parasite load. Pikas showed increased boldness, exploration, and docility on day 5, when *Eimeria* spp. numbers were high. The T3 and T4 levels declined as parasite loads increased, whereas the CORT and RMR levels fluctuated at different experimental stages. These results suggest that parasite infection influences host behavior and physiology, providing insights into parasite-host interactions.

**Conclusion:**

This study provides evidence that *Eimeria* spp. load modulates the behavior and physiology of plateau pikas. The specific behavioral shifts coinciding with peak infection, coupled with the suppression of thyroid hormones and metabolic responses, reveal a complex and integrated host adaptation strategy. As a keystone species on the Qinghai-Tibet Plateau, the behavior-physiology coupling mechanism in plateau pikas not only provides new evidence for host-parasite coevolution, but also offers critical insights into understanding the stability of regional ecosystems.

## Introduction

Parasites are ubiquitous among vertebrates and play important roles in ecosystems (Zelmer, 1998). They usually have nonlethal effects on their hosts, but can sometimes lead to host mortality by altering or manipulating host behavior, physiology, or other phenotypic traits (McElroy & De Buron, 2014; Hernandez-Caballero et al., 2022; Jensen et al., 2023; Piquet et al., 2018). Unlike large predators, parasites typically do not cause host death directly but rather significantly affect host physiology and behavior through persistent energy consumption (Barber, 2007). The extent of parasite impact depends on many factors, including parasite number, infection intensity, host body condition, and developmental stage (Gartland et al., 2022; Krist et al., 2004). For example, bridled goby (*Coryphoptererus glaucofraenum*) infected with parasites may exhibit increased boldness behavior or reduced predator defense capabilities, thereby facilitating parasite spread (Forrester et al., 2019). Infection with *Toxoplasma gondii* increased exploration in brown rats (*Rattus norvegicus*) (Vyas et al., 2007). Additionally, parasitic infection reduces fecal cortisol concentrations in reindeer (*Rangifer tarandus*) (Carlsson et al., 2016) while increasing cortisol concentrations in red colobus monkeys (*Colobus polykomos*) (Snaith et al., 2008). Long-term infections can also shorten host lifespan, reduce fertility, and even impact population fluctuations (Adamo, 2013; Barber & Dingemanse, 2010).

Animal personality refers to the relatively consistent behavioral differences exhibited by individuals across contexts (Réale et al., 2007; She et al., 2022). It has been demonstrated that physical condition plays a crucial role in shaping animal personality (Dingemanse & Wolf, 2010), while parasitic infections influence personality by affecting physical condition (Dunn, Cole & Quinn, 2011), thereby improving transmission efficiency (Barber & Dingemanse, 2010). Infected animals often exhibit behavioral changes such as decreased activity, foraging (Santicchia et al., 2020), and social interactions due to decreased physical strength (*i.e*., weight loss) and heightened immune responses (*e.g*., elevated cortisol levels and increased inflammatory factors), which may represent host strategies to conserve energy and avoid further pathogen exposure (Lopes, Block & König, 2016; Moore, 2002). For example, mice infected with nematodes spend less time foraging, but more time resting, thereby conserving energy to supply their immune system to defend against infection (Hayes et al., 2010). Additionally, host exploration and aggressiveness may be reduced after infection by parasites, which in turn affects critical survival behaviors, such as predation and territorial defense (Binning, Shaw & Roche, 2017; Tong et al., 2021). Parasite infection also stimulates the release of stress hormones, for instance cortisol, by triggering the host’s hypothalamic-pituitary-adrenal (HPA) axis. Elevated cortisol levels can directly affect host behavior, such as decreasing willingness to explore new environments and reaction times (Allan et al., 2020; Rusch, Layden & Dugas, 2023). Recently, numerous studies have focused on the effects of parasitic infections on animal personality, providing new insights into host-parasite interactions. Parasites can alter host behavior not only by directly affecting physiological functions but also by shaping the host personality through long-term behavioral feedback (Ezenwa et al., 2016; Poulin, 2013). These dynamic relationships profoundly impact hosts’ health and survival, and further influence their behavioral strategies and adaptations over the evolutionary process. Studying the relationship between parasite infection and personality will contribute to revealing the physiological basis of individual behavioral differences in animals, providing an important theoretical foundation for disease ecology, wildlife conservation, and management.

*Eimeria* spp. are a group of specialized intracellular parasitic protozoa that are widely parasitized in the intestinal tract of vertebrates and frequently cause coccidiosis in their hosts (Chapman, 2014; Shirley, Smith & Tomley, 2005). These parasites survive in the host through asexual and sexual reproduction stages, culminating in the formation of oocysts excreted in feces and transmitted into the environment (Singla, Gupta & Lather, 2012; Williams, 2001). *Eimeria* spp. induce inflammatory responses primarily by destroying gut epithelial cells (Bangoura & Daugschies, 2017; Shirley, Smith & Tomley, 2005), ultimately resulting in reduced nutrient absorption capacity and subsequently inducing physiological stress in the host, with principal manifestations including decreased body mass, metabolic disorders, and decreased energy availability (Attree et al., 2021; Bangoura & Daugschies, 2017). For example, wild red colobus monkeys (*Procolobus rufomitratus tephrosceles*) infected with *Trichuris* spp. exhibit reduced activity and energy metabolism (Ghai et al., 2015). Additionally, parasitic infections stimulate the host immune response, elevating stress hormone concentrations such as cortisol, which contributes to short-term metabolic regulation but may suppress immune function and further exacerbate infection over the long term (Attree et al., 2021; Gao et al., 2024). Thyroid hormones are key regulators of basal metabolic rate and reflect the overall energy expenditure status of the host. Parasitic infections can suppress host metabolism by restraining thyroid hormone levels, reducing energy expenditure, and prioritizing supply to the immune system (Rauw, 2012; Réale et al., 2007; Robar, Murray & Burness, 2011). According to the energy partitioning model, when the host responds to parasites by preferentially allocating energy to the immune system, the energies used for other functions, such as growth and reproduction would decrease (Hammond & Diamond, 1997; Réale et al., 2007). Thus, parasitic infections may influence the host energy metabolism through multiple pathways, triggering a range of physiological changes.

Plateau pika (*Ochotona curzoniae*) is an important small herbivore widely distributed in the Qinghai-Tibetan Plateau (Smith & Foggin, 1999; Yu et al., 2012). They play important ecological roles by regulating grassland ecosystem biodiversity and function (Zhao et al., 2020). However, when population density is too high, pika digging and foraging may aggravate grassland degradation (Pech et al., 2007; Wei et al., 2023). Consequently, regulating the population abundance of plateau pika is essential for maintaining alpine meadow ecosystem balance on the Qinghai-Tibetan Plateau (Zhou et al., 2023). As a primary parasite of plateau pikas, *Eimeria* spp. are potential biological control agents (Bian et al., 2011). *Eimeria* spp. have been shown that *Eimeria* spp. may influence pika population dynamics (Bian et al., 2011; Du et al., 2012). For instance, infection with *Eimeria* spp. significantly increases pika mortality (Bian et al., 2011) and reduces pika fertility (Yanbin et al., 2015). However, the relationships between *Eimeria* spp. and pika behavior and physiology remain unclear. In this study, we established three treatment groups: *Eimeria* spp. (PA+), normal saline (Ctrl), and anticoccidials (PA−), to explore the effects of *Eimeria* spp. on personality (*i.e*., exploration, boldness, and docility) and physiology (*i.e*., resting metabolic rate (RMR), cortisol (CORT), serum triiodothyronine (T3), and thyroxine (T4) concentrations). We hypothesized that (1) *Eimeria* spp. numbers fluctuate with the infection cycle; (2) because of the stress of parasitic infection, pikas become less explorative and shy, but more docile to balance the energy consumption of normal activity and immune response; and (3) high-intensity infections increase the cortisol levels of pikas while reducing energy metabolism and thyroid hormone concentrations. This study not only provides evidence for a better understanding of host-parasite coadaptation and coevolution but also offers a potential and efficient alternative for the management of small mammal pests.

## Materials and Methods

### Animal capture and housing condition

A total of 30 healthy adult plateau pikas (138.61 ± 8.08 g) with a uniform genetic background were captured in September 2020 from a single area (37° 56′N, 101° 4′E, 3,062 m) within the alpine meadow of Haibei Prefecture, Qinghai Province. To ensure the selection of healthy, reproductively mature adult animals suitable for the experiment, animals were included only if they met the following criteria: specifically, they were classified as adults based on body weight (>130 g); showed no signs of external injuries, overt illness, or pregnancy; and exhibited normal activity and foraging behavior upon capture. After capturing the animals, we examined the feces of all individuals and confirmed the presence of *Eimeria* oocysts under natural infection conditions. The pikas were individually housed in polypropylene cages (450 × 289 × 180 mm) under a natural light cycle at the laboratory of the Northwest Institute of Plateau Biology, Chinese Academy of Sciences. The ambient temperature and humidity were set to natural levels, with the temperature maintained at 22 ± 2 °C and the humidity at 60 ± 5%. These conditions were consistent throughout the experiment, with no significant fluctuations during the study period. The cages were cleaned and the padding was changed regularly. Before the experiment commenced, the animals were acclimated to the laboratory environment for 7 days and fed a standard laboratory rabbit diet (Beijing Ke Ao Food, Co., Beijing, China) and water *ad libitum*.

This study was conducted in accordance with protocols approved by the Ethics Committee of the Northwest Institute of Plateau Biology, Chinese Academy of Sciences (Approval No. 2020-15).

### Experimental group

Based on the resource equation approach for intergroup comparison using analysis of variance (ANOVA), the minimum sample size required per group in this study was calculated to be five animals. This method relies on the principle that the error degrees of freedom should lie between 10 and 20 to ensure sufficient statistical power for the model (Arifin & Zahiruddin, 2017). Given that the experimental animals were wild-caught and considering the potential risk of mortality associated with parasitic infection treatments, the sample size was conservatively increased to 10 animals per group. This adjustment ensures that the final number of animals per group exceeds the maximum estimated requirement of seven, derived from the resource equation approach, thereby maintaining sufficient statistical power even if individual dropouts or mortality occur during the experiment.

To explore the effects of parasitic infection on the physiology and behavior of plateau pikas, the experiment was designed with three treatment groups: (1) *Eimeria* infection group (PA+), (2) a control group with normal saline gavage (Ctrl), and (3) an anticoccidial drug-treated group (PA−). The experimental design aimed to compare the physiological and behavioral responses of hosts under different levels of parasite load. Animals in the PA+ group were fed *Eimeria* oocysts to simulate a high-parasite-infection condition. Animals in the Ctrl group were fed physiological saline to represent the baseline state under natural physiological conditions. Animals in the PA– group were fed an anticoccidial drug to inhibit *Eimeria* reproduction, thereby mimicking a low-parasite-load condition. Plateau pikas were randomly allocated into one of three treatment groups (*n* = 10 per group) using a random number generator (Microsoft Excel, Microsoft, Redmond, WA, USA). Before the treatments began, the number of *Eimeria* oocysts in the feces of all plateau pikas was assessed. Treatment was only initiated when the *Eimeria* oocyst count in each group ranged between 2,500 and 3,000 and there were no significant differences in oocyst numbers among the groups. This ensured that at the start of the experiment, the oocyst load was comparable across all three groups, thus maintaining consistency in initial conditions.

In the PA+ group, pikas were fed 2.0 × 10^6^ PCS/mL of *Eimeria* spp. oocysts, as it has been shown that the cleavage reproduction of *Eimeria* mainly destroys the intestinal tissues of the host, and when 4 × 10^6^ Eimeria/mL^−1^ were fed to plateau pikas, the death of plateau pikas appeared on the 4th day, and the death rate of plateau pikas reached the maximum on the 8th day (Yang, 2017). The Ctrl group received 1 mL of normal saline, while the PA− group was treated with anticoccidial drugs. All treatments were administered *via* oral gavage using a rat-sized stainless steel gavage needle (diameter 1.2–1.6 mm, length 65–80 mm). Animals need to be fasted for 6–8 h before gavage (free access to water), animals were gently restrained with head and neck maintained in a straight line. Before insertion, the needle tip was smoothed if overly sharp. The needle was inserted gently from the left corner of the mouth along the hard palate into the esophagus. The needle was inserted to an appropriate depth, and the solution was injected only after confirming no air reflux upon withdrawal of the syringe. During the gavage procedure, if the pika exhibited vigorous struggling or a vomiting reflex, the needle was immediately withdrawn to prevent tracheal injury. The solution was administered at a smooth and steady rate to avoid choking or reflux caused by overly rapid delivery. Successful administration was confirmed by normal breathing after release. The main ingredient of the anticoccidial drug was sodium sulfamethoxazole, at a dosage of 0.0012 × the body mass of each pika (Noack, Chapman & Selzer, 2019).

The experiment was divided into three stages: day 5 (D5), day 8 (D8), and day 18 (D18), based on the *Eimeria* immitis pathogenicity cycle. During these stages, personality and physiological characteristics of plateau pikas were measured. Subsequently, the metabolic rate and behaviors (*i.e*., exploration, boldness, and docility) were assessed on D5, D8, and D18 after treatment. Fresh fecal samples were collected from each individual on D5, D8, and D18, and their physiological traits (*i.e*., T3, T4, and CORT concentrations as well as *Eimeria* spp. numbers) were tested. This resulted in a final sample size of *n* = 10 for each experimental group (PA+, Ctrl, PA−) for all subsequent behavioral, physiological, and statistical analyses. All individuals completed the entire study protocol, and data were collected from all subjects at all three time points (D5, D8, D18).

The experiment was conducted in a controlled laboratory environment to minimize external variability. To control for potential confounding factors, the order in which animals from different groups were processed (*e.g*., gavage, behavioral testing) and measured each day was randomized; furthermore, the sequence of behavioral tests (exploration, boldness, docility) was counterbalanced across individuals to avoid order effects. The housing cages for all three groups were intermixed and randomly assigned within the animal room to prevent systematic spatial differences in environmental conditions (*e.g*., light, noise, human activity) from being confounded with treatment effects. During the test, the ambient temperature and humidity were kept stable at approximately 22 ± 2 °C and 60 ± 5% relative humidity. This control minimized potential effects from the subjects or the oocysts they excreted. To maintain hygiene without introducing external disturbances, cages were replaced daily and cleaned with boiling water. Following the experiments, the animals were euthanized *via* inhalation of an overdose of isoflurane anesthesia. All animal procedures were approved by the Animal Protection and Use Committee of the Northwest Plateau Institute of Biology, Chinese Academy of Sciences. According to the 3R principle of animal welfare, efforts were made to minimize the number of animals used and reduce their suffering.

### Collection and cultivation of *Eimeria* spp.

*Eimeria* spp. were collected and cultured in a laboratory. Collect fecal samples from plateau pikas infected with *Eimeria* spp., feces were homogenized with purified water, and 0.8% sodium chloride solution with a volume of 5 mL was combined with 2 g of the prepared feces mixture. After 5 min of centrifugation at 3,000 rpm, the sediment was collected and *Eimeria* spp. oocysts were isolated using saturated saltwater floatation. Subsequently, the oocysts were treated with a 2.5% potassium dichromate solution and incubated at 27 °C to induce sporulation (Bian et al., 2011). After multiple rounds of oocyst proliferation, *Eimeria* oocysts were obtained.

### Determination of *Eimeria* oocyst count

*Eimeria* spp. in feces were calculated using McMaster’s method (Du et al., 2012). We took 2 g of fecal sample and added 20 mL of saturated saline solution, then mixed it well. We filtered the mixture sequentially through 0.425 mm and 0.150 mm sieves. We took 1 mL of the filtrate and mixed it with 9 mL of water. This dilution step was intended to facilitate the counting of oocysts in the McMaster chambers by ensuring the oocysts were adequately separated and distributed for more accurate counting. After thorough mixing, we used a capillary pipette to withdraw a small amount of the diluted liquid and transfered it into the counting chambers of a McMaster counting slide. We placed the slide on the microscope stage, let it stand for a few minutes, and then count all *Eimeria* oocysts in both chambers under low magnification. Finally, we took the average count and convert it to the number of oocysts per gram of feces.

### Anticoccidial drug

In the present study, sodium sulfachyloropyrazine was used to suppress *Eimeria* spp. The active components of sodium sulfachloropyrazine and sulfanilamide compete with cyanobenzoic acid for dihydrofolate synthetase, thereby disrupting the synthesis of dihydrofolate and nuclear proteins (Li et al., 2024; Zhang et al., 2012). Drug efficacy was assessed using the anticoccidial index (ACI) with the following evaluation criteria: scores of 120 or less were deemed ineffective, values between 120 and 160 indicated low efficiency, ratings from 160 to 180 suggested medium efficiency, and results exceeding 180 indicated high efficiency (Li et al., 2024). The ACI range for sodium sulfachloropyrazine in preventing and treating intestinal coccidia is 147–197, indicating the effectiveness of this compound in combating coccidia (Li et al., 2024; Xue et al., 2012).

### Behavioral measurements

Three types of behavior in plateau pikas were assessed: exploration, boldness, and docility (Qu et al., 2018). An open-field arena with a bottom area of 50 × 50 cm was used to measure exploration (Careau et al., 2015). Considering the activity rhythm of the species, the measurements were conducted between 8:00 and 11:00 a.m. The central area was a 40 × 40 cm square at the center of the bottom, while the remaining peripheral area was designated as the edge. After an individual was placed in the arena, its trajectory was recorded using a video camera and the EthoVision IX animal tracking system was used for behavioral analysis. Specifically, the moving distance of an animal within 180 s in the open field was analyzed, indicating the exploration of pikas (Zhu et al., 2022). After each measurement, wiping residual urine, hair and feces from the field with 75% alcohol to prevent interference with subsequent experiments.

After the exploration test, an 18 × 10 × 12 cm concealment box was randomly placed in one corner of the open-field arena. The center bottom of the arena had an opening facing upwards. Once an individual quickly entered the refuge, the time it first left the refuge within 2 min was recorded as boldness. If the individual did not leave the refuge within 2 min, the boldness was considered 120 s (Cheng et al., 2023; Réale et al., 2007). Across all 30 individuals tested three times each (90 trials in total), 11 trials (12.2%) resulted in the maximum latency of 120 s, indicating that most pikas exited the refuge within the test duration. Therefore, the boldness test was generally effective in capturing inter-individual differences in this species. The latency to leave the refuge was inversely related to the boldness value. Subsequently, a mesh bag was used to determine the struggling response of each individual, which represented the docility of the pikas. Each individual was suspended in a nylon mesh bag, and the time the individual remained stationary for 1 min was recorded as docility (Réale et al., 2007). The level of docility increased with time spent stationary. All individuals were tested for their behavior at three different time points, with the behavioral tests conducted in the consistent order of exploration, boldness, and docility, and all behaviors were measured on the same day for each time point.

### Resting metabolic rate measurement

A portable FMS (Sable Systems International, Henderson, NV, USA) was used to determine the resting metabolic rate (RMR) of plateau pika. The RMR measurements were conducted after the completion of the three behavioral tests, between 8:00 and 11:00 a.m., corresponding to the morning active period of plateau pikas to minimize the potential influence of circadian rhythm on metabolism. The respirometry system consisted of eight transparent respiratory chambers maintained at 27 ± 2 °C, which represents the thermoneutral zone of plateau pikas. Chamber No. 1 served as a baseline control for oxygen (O_2_), carbon dioxide (CO_2_), and water vapor, while each of the remaining seven chambers housed one pika. Thus, the RMR of seven individuals could be measured simultaneously (Zhu et al., 2022).

Following the docility test, each pika was gently transferred into a respiratory chamber and allowed to acclimate for at least 30 min to ensure it reached a calm and resting state. After acclimation, RMR recording began. Each complete measurement session lasted for 2 h, during which RMR was continuously recorded in four consecutive 30-min cycles to ensure data stability and repeatability. Each measurement cycle was automatically repeated ten times within the 2-h period to reduce random variation and improve measurement accuracy (Zhu et al., 2022).

### Hormone concentration measurement

Fecal samples were obtained immediately after excretion and maintained at −20 °C in a freezer, and the CORT, T3, and T4 concentrations were measured (Mortavazi et al., 2009; Yu et al., 2021). Fecal samples were thoroughly mixed with a glass rod and 10% fecal homogenates were prepared by combining 1 g of feces with 9 mL of saline. Each homogenate was centrifuged at 3,000 r/min for 15 min, and 1 mL of the supernatant was collected (Zhao et al., 2018). Hormone (CORT, T3, and T4) concentrations were quantified using an enzyme-linked immunosorbent assay (ELISA) kit (Guangzhou Leizhi Biotechnology Co. Ltd., Guangzhou, China). The assay exhibited a detection threshold of 1.0 ng/mL, while displaying 15% variability for both intra-assay and inter-assay measurements. All hormone concentration were measured on the same day for each time point.

### Statistical analysis

No animals or data points were excluded during the experiment or from the subsequent analysis. All 30 captured individuals that met the inclusion criteria were randomly allocated into the three experimental groups (*n* = 10 per group), and all collected data were included in the final statistical analyses. All statistical analyses were conducted using the R software (v. 4.4.2). In animal behavior research, repeatability is commonly used to measure individual behavioral consistency. In this study, the “*rptR*” package was used to assess the repeatability of behavioral and physiological traits (Stoffel, Nakagawa & Schielzeth, 2017), with treatment as a fixed effect and individual ID as a random effect. Confidence intervals (CIs) were estimated using the bootstrap method, with 1,000 iterations (nboot = 1,000), and the number of permutations for the randomization test (npermut) was also set to 1,000 (Stoffel, Nakagawa & Schielzeth, 2017). Behavioral and physiological traits were modeled using a Poisson distribution. Unadjusted repeatability was estimated by fitting a mixed model with treatment as a fixed effect and individual ID as a random effect. This estimate reflects the proportion of variance explained by individual differences. To obtain adjusted repeatability estimates, measurement time and gender were added as fixed effects to control for their potential influence on the measurements. The adjusted repeatability reflects the proportion of variance explained by individual differences after controlling for time and gender effects. The final results are presented as both unadjusted and adjusted repeatability estimates, along with 95% confidence intervals.

The “Shapiro-Wilk test” was employed to evaluate the normality of data and “Bartlett’s test” was employed to evaluate the homogeneity of variance across groups, which revealed that the data on behavioural and physiological traits were non-normally distributed (Mollan et al., 2020). Since the data did not satisfy normality, we used the Mann-Whitney U test to compare animal personality and physiology among the three experimental groups (Mollan et al., 2020). Data were subjected to logarithmic transformation for graphical representation. To evaluate the potential effects of *Eimeria* spp., anticoccidial drug, and saline on personality and physiological characteristics, we employed linear mixed-effects models (LMMs). We used a linear mixed-effects model (LMM) to analyze the changes in the relative importance of the effects of treat, time and sex on animal behaviors and physiological traits (West, Welch & Galecki, 2022). The model explained the proportion of treat, time, sex and their interactions explained, where the main effect is (1) treatment effect (PA+ group *vs* PA− group *vs* Ctrl group), (2) time effect (*i.e*., D5, D8, and D18), (3) sex effect. The interaction effect is (1) between treatments and time, ID as a random effect. We used the “lem4” package to fit the LMM. A hierarchical split for the proportion of explanations in each section within each section (*P* values) was performed using the “*glmm.hp”* package (Lai et al., 2023, 2022).

The analysis of behavioral syndromes entailed the application of prior distributions and mixed-effects multivariate models. Covariances and correlations between personality pairs were estimated utilizing the “*MCMCglmm”* package, which also facilitated the detection of behavioral syndromes. Prior distribution parameters were established with an expected variance V = diag(2) and a degree of belief nu = 1.002 (Polverino et al., 2023; Wang et al., 2024). Multivariate mixed models treat each combination of personality and physiological variables as the dependent variables. These models were executed for 1,500,000 iterations, incorporating a 500,000 iteration burn-in phase and thinning interval of 100 iterations (Polverino et al., 2023; Wang et al., 2024). Following model execution, posterior probability distribution plots were examined using the plot function to confirm the appropriate model mixing and convergence. Variances were subsequently calculated for within-individual, among-individual, and phenotypic factors (Polverino et al., 2023; Wang et al., 2024). Phenotypic variance is subdivided into among-individual variance (indicative of behavioral syndromes) and within-individual variance (representing plasticity integration). Phenotypic correlations emerged from a combination of within-individual and among-individual correlations. The results were presented as correlation estimates with 95% credible intervals, with significance determined by the non-overlap of these intervals with zero (Polverino et al., 2023; Wang et al., 2024).

## Results

### Repeatability of physiology and behavior

Both the personality and physiological traits of plateau pikas exhibited moderate repeatability. The repeatability estimates for exploration and docility showed comparable patterns (Radj = 0.326 [0.108, 0.557] and Radj = 0.326 [0.137, 0.553], respectively). The lowest repeatability for RMR was 0.299 [0.071, 0.574] and the highest repeatability for boldness was 0.328 [0.121, 0.564]. The average repeatability was 0.319 [0.108, 0.567] (Table 1).

**Table 1:** Repeatability analyses result for each behavior and physiology traits (*N* = 90 tests from 30 individuals). Unadjusted and adjusted repeatability (R) estimates with 95% credible intervals.

Variables	Unadjusted R	Adjusted R
T3	0.312 [0.090, 0.583]	0.308 [0.096, 0.571]
T4	0.333 [0.135, 0.614]	0.327 [0.126, 0.573]
CORT	0.322 [0.120, 0.608]	0.322 [0.100, 0.589]
RMR	0.299 [0.073, 0.598]	0.299 [0.071, 0.574]
Exploration	0.333 [0.126, 0.572]	0.326 [0.108, 0.557]
Boldness	0.331 [0.135, 0.612]	0.328 [0.121, 0.564]
Docility	0.329 [0.112, 0.590]	0.326 [0.137, 0.553]

**Note:**

R-values are adjusted repeatability including the fixed effect of the test version. *P* values and 95% confidence intervals (in brackets) are based on 1,000 bootstrapping rounds. The original scale approximations for Poisson models have been reported.

### Variations in the *Eimeria* spp. number

**Figure 1:**
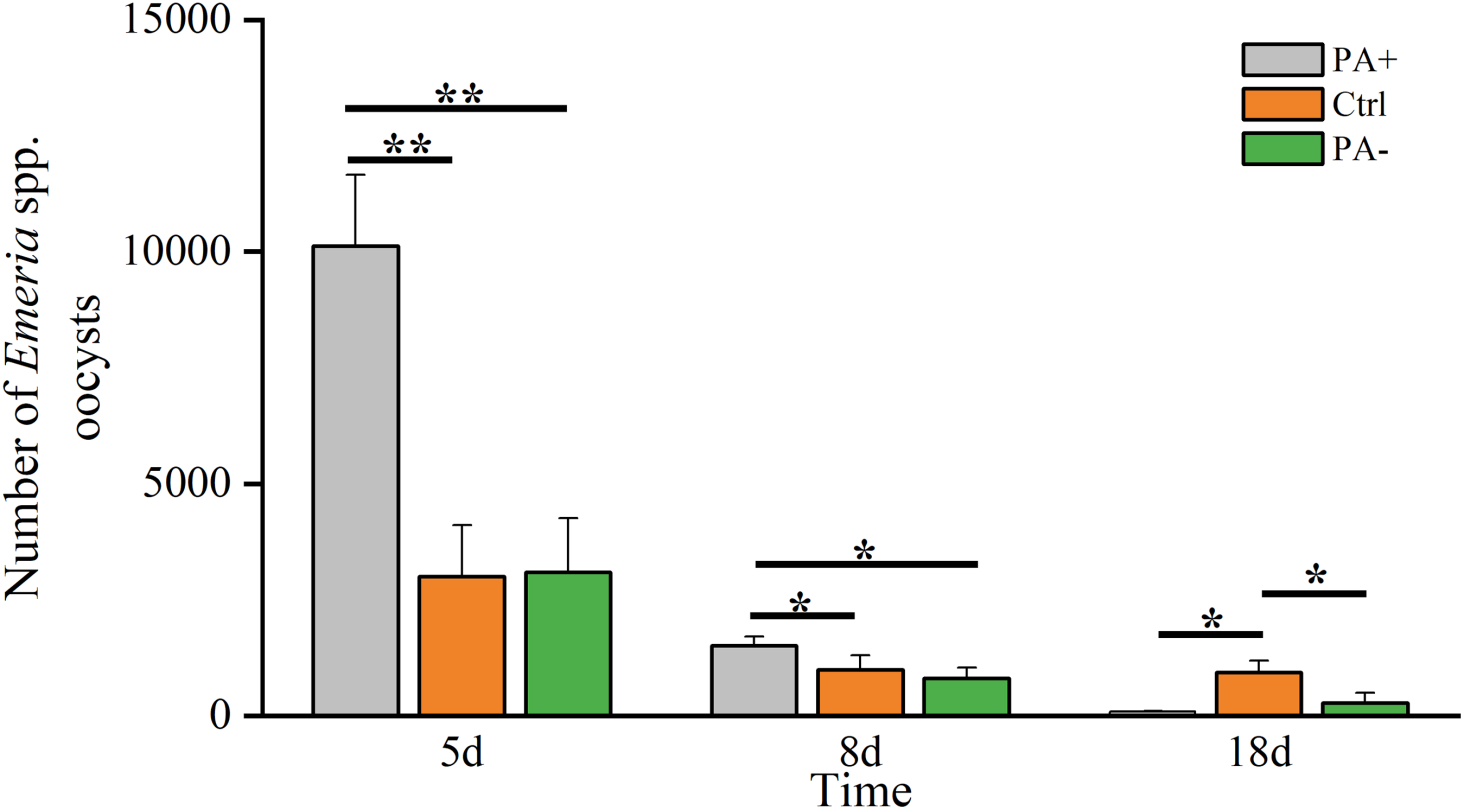
Difference in the *Eimeria* spp. number in plateau pikas from the three treatment groups on D5, D8, and D18. Note: PA+ indicates feeding *Eimeria* spp. oocysts group, Ctrl indicates control group, and PA− indicates anticoccidial group. Asterisks indicate significant differences among different treatment groups at the same time: *P* < 0.05 (*) and *P* < 0.01 (**). This also applies to the following figures.

The *Eimeria* spp. decreased over time (Fig. 1, Table 2). On D5, *Eimeria* spp. peaked in each group, with the PA+ group was significantly higher than that in the Ctrl and PA− groups (*P* = 0.005, *P* = 0.008). On D8, *Eimeria* spp. in the PA+ group remained significantly higher than in the Ctrl and PA− groups (*P* = 0.039, *P* = 0.033). However, the *Eimeria* spp. numbers in the Ctrl group were significantly higher than those in the PA+ and PA− groups on D18 (*P* = 0.017, *P* = 0.026).

**Table 2:** Descriptive statistics of *Eimeria* spp. Number, personality and physiological traits across experimental groups and time points.

Variable	Time	PA+	Ctrl	PA−
*Eimeria* spp. number	D5	10,114.97 ± 1,541.23	3,001.63 ± 1099.90	3,085.50 ± 1,173.92
	D8	1,505.71 ± 197.27	985.71 ± 311.30	810.72 ± 226.00
	D18	88.57 ± 17.92	927.57 ± 259.79	275.87 ± 212.73
Exploration (mm)	D5	198.27 ± 47.91	512.47 ± 257.66	373.50 ± 109.88
	D8	431.04 ± 150.81	1,457.38 ± 711.80	354.62 ± 110.79
	D18	372.78 ± 83.41	115.76 ± 53.60	15.79 ± 4.87
Boldness (s)	D5	21.34 ± 3.36	51.60 ± 16.16	15.46 ± 4.78
	D8	33.71 ± 7.76	61.43 ± 16.54	30.54 ± 3.76
	D18	65.48 ± 14.55	67.11 ± 11.51	31.09 ± 7.81
Docility (s)	D5	25.50 ± 4.79	15.90 ± 4.93	20.70 ± 4.86
	D8	31.20 ± 6.71	19.60 ± 5.46	22.07 ± 3.33
	D18	39.50 ± 5.71	40.19 ± 6.17	37.05 ± 5.58
T3 (nmol/L)	D5	4.36 ± 0.23	3.05 ± 0.15	5.85 ± 0.20
	D8	3.93 ± 0.16	3.32 ± 0.24	5.70 ± 0.21
	D18	4.22 ± 0.18	4.04 ± 0.21	6.12 ± 0.23
T4 (nmol/L)	D5	151.89 ± 5.73	89.70 ± 3.53	170.96 ± 6.77
	D8	153.09 ± 4.55	130.74 ± 5.84	198.56 ± 5.42
	D18	153.09 ± 4.55	110.88 ± 6.85	187.55 ± 5.93
RMR [ml/(g.h)]	D5	2.45 ± 0.20	2.15 ± 0.28	1.56 ± 0.30
	D8	2.57 ± 0.32	2.15 ± 0.52	2.39 ± 0.46
	D18	2.38 ± 0.13	3.39 ± 0.11	3.12 ± 0.13
CORT (ng/ml)	D5	8.88 ± 0.40	8.46 ± 0.61	9.79 ± 0.50
	D8	8.21 ± 0.32	9.71 ± 0.52	6.40 ± 0.15
	D18	9.92 ± 0.56	7.67 ± 0.40	10.02 ± 0.33

**Note:**

Data are showed as means ± S.E.

### Variations in personality

**Figure 2:**
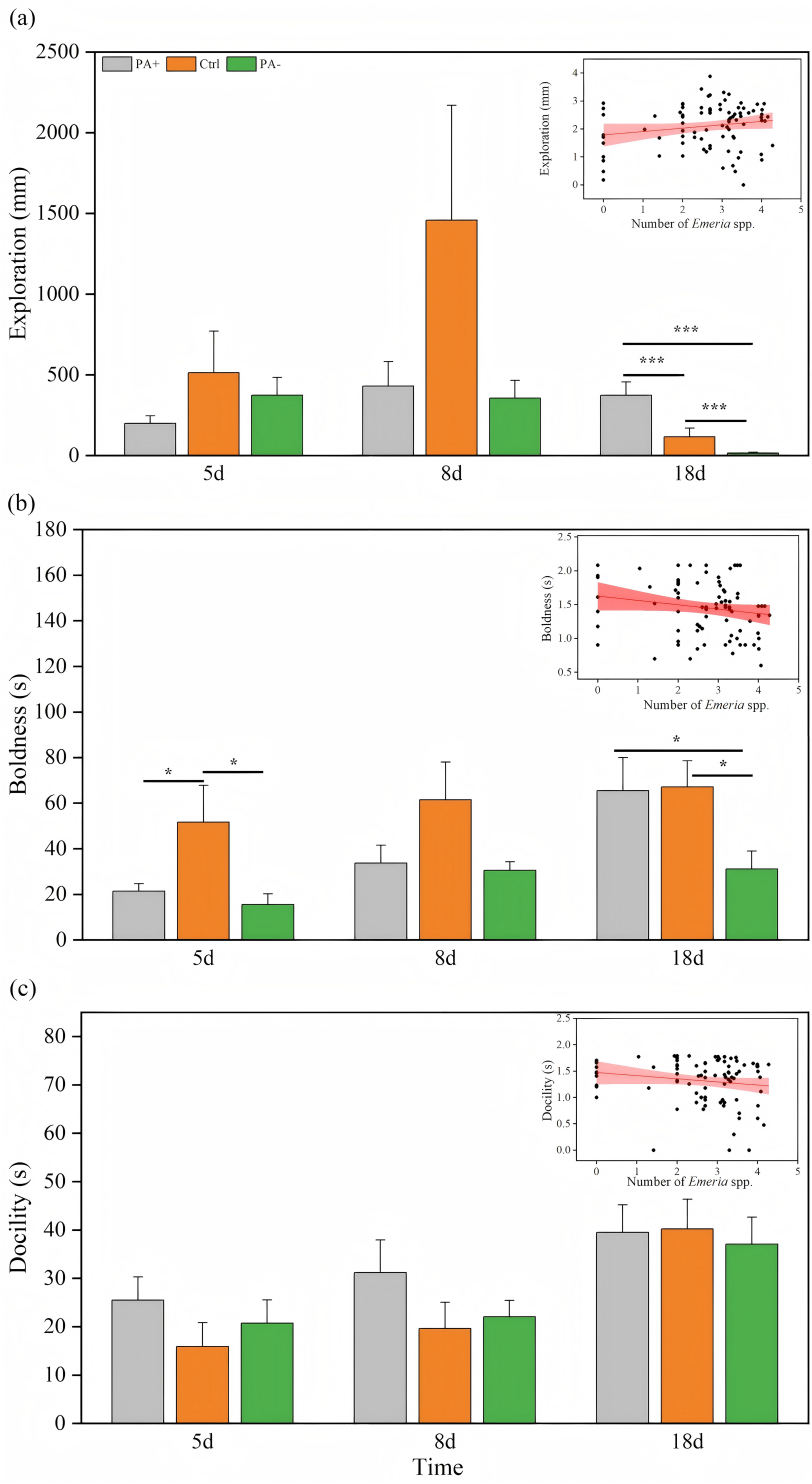
Comparison of exploration, boldness, and docility across different treatment groups on D5, D8, and D18. The insets show the relationship between personality traits and *Eimeria* spp. count, respectively, with the fitted line and its red shaded area representing the regression analysis and the 95% confidence interval. (A) Exploration: The distance (mm) the pika travels in the open field test. (B) Boldness: The time (s) it takes for the pika to emerge from the refuge. (C) Docility: The amount of time (s) the pika spends resting in the mesh bag. Notes: Inset plots show the fitted relationships between personality (exploration, boldness, docility) and *Eimeria* spp. All points represent raw data collected from all treatment groups (PA+, Ctrl, and PA−) and time points (D5, D8, and D18), and the fitted lines were generated from these observations rather than predicted values from the MCMCglmm models. Bar plots are also based on raw data (mean ± SE) to ensure transparency and accurately reflect observed variation. Asterisks indicate significant differences among different treatment groups at the same time: *P* < 0.05 (*) and *P* < 0.001 (***).

Time significantly explained the variation in exploration (*P* = 0.024, Table 3). As time increased, the PA+ and Ctrl groups showed inverted parabolic trends in exploration, whereas exploration in the PA− group decreased over time (Fig. 2A, Table 2). Exploration of the Ctrl and PA− groups was higher than that of the PA+ groups on D5. On D8, exploration was higher in the Ctrl group than that in the PA+ and PA− groups. On D18, exploration in the PA− group was significantly lower than that in the Ctrl and PA+ groups (*P* = 0.001, *P* = 0.001, Fig. 2A, Table 2). Overall, with an increase in *Eimeria* spp., pikas became more exploratory. Specifically, from D5 to D18, as *Eimeria* spp. decreased in each group, the PA− group showed the highest exploration on D5, whereas the Ctrl and PA+ groups exhibited the highest exploration on D8.

**Table 3:** Results from the LMM analyses with treat, time and sex as fixed effects, and ID as a random effect. The last column reports the marginal R^2^ with 95% confidence intervals of the model (row: fixed effects) and the variance explained by Treat, time, sex and interaction (reported as semi-partial R^2^; see Methods for details). The bold entries indicate statistically significant results (*P* values < 0.05).

Variables	Fixed effects	DenDF	F value	*P* value	Deviation explained (%)
T3	Treat	25.74	104.89	**<0.001**	46.84
	Time	53.66	4.87	**0.011**	1.760
	sex	28.08	1.75	0.196	0.680
	Treat ×Time	53.74	2.22	0.078	50.71
T4	Treat	80.00	139.66	**<0.001**	43.59
	Time	80.00	13.35	**<0.001**	4.190
	sex	80.00	1.57	0.213	1.150
	Treat × Time	80.00	3.46	**0.012**	51.07
CORT	Treat	80.00	0.61	0.548	0.870
	Time	80.00	5.39	**0.006**	8.740
	sex	80.00	1.12	0.294	1.730
	Treat × Time	80.00	12.52	**<0.001**	88.66
RMR	Treat	26.11	0.23	0.794	1.160
	Time	53.99	8.50	**<0.001**	29.02
	sex	29.66	0.06	0.810	−0.60
	Treat × Time	54.10	3.25	**0.018**	70.43
Exploration	Treat	26.08	2.05	0.148	12.02
	Time	53.10	3.98	**0.024**	19.57
	sex	28.63	0.09	0.765	0.940
	Treat × Time	54.08	2.04	0.101	67.48
Boldness	Treat	26.09	4.85	**0.016**	28.42
	Time	53.93	5.94	**0.005**	14.61
	sex	30.69	1.77	0.194	5.710
	Treat × Time	54.07	1.14	0.347	51.26
Docility	Treat	26.09	0.75	0.484	5.500
	Time	53.89	16.08	**<0.001**	41.21
	sex	32.10	0.65	0.427	3.150
	Treat × Time	54.07	0.66	0.620	50.14

Time also had significant effects on boldness and docility (*P* = 0.005, *P* = 0.0001, Table 3). With increasing experimental time, both boldness and docility showed increasing trends in different groups (Figs. 2B and 2C). On D18, boldness was significantly lower in PA− group than in PA+ and Ctrl groups (*P* =0.021, Fig. 2B, Table 2), there was similar trends on D8 but without statistical significance.

For docility, as time increased, the Ctrl group exhibited the highest docility on both D5 and D8, followed by the PA− and PA+ groups. On D18, the PA− group had higher docility than the Ctrl and PA+ groups (Fig. 2C, Table 2). Overall, plateau pikas exhibited greater boldness with higher *Eimeria* spp. The Ctrl group exhibited the lowest boldness followed by the PA+ and PA− groups. Additionally, as *Eimeria* spp. increased, plateau pikas exhibited greater docility, with the PA+ group exhibiting the lowest docility among the three groups.

### Variations in physiological traits

**Figure 3:**
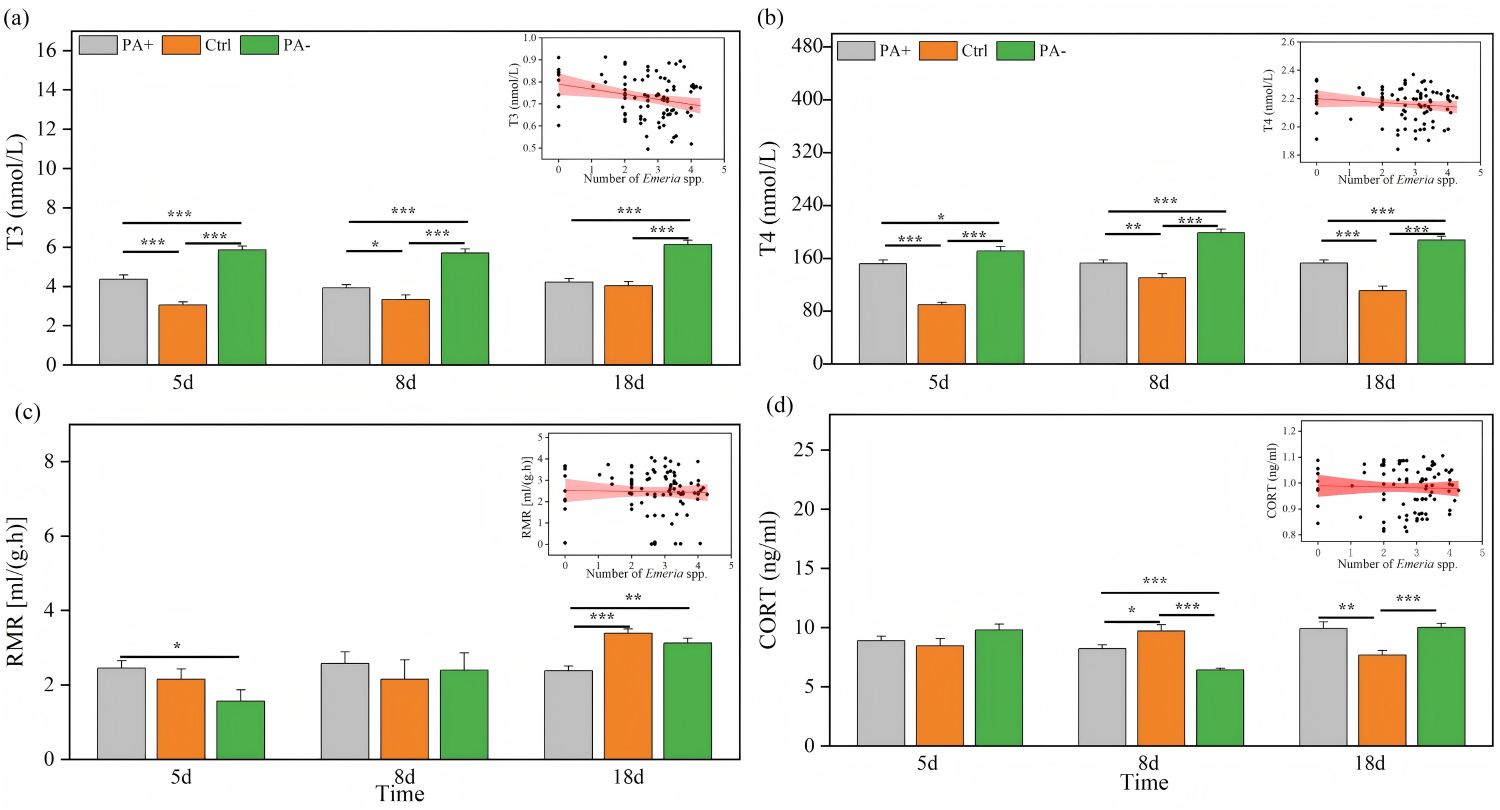
Comparison of the physiological traits of plateau pikas across different treatment groups on D5, D8, and D18. The insets show the relationship between physiological traits and *Eimeria* spp. count, respectively, with the fitted line and its red shaded area representing the regression analysis and the 95% confidence interval. (A) T3: Serum triiodothyronine concentration. (B) T4: Thyroxine concentration. (C) CORT: Cortisol concentration. (D) RMR: Resting metabolic rate level. Notes: Inset plots show the fitted relationships between physiological traits (T3, T4, CORT, and RMR) and *Eimeria* spp. All points represent raw data collected from all treatment groups (PA+, Ctrl, and PA−) and time points (D5, D8, and D18), and the fitted lines were generated from these observations rather than predicted values from the MCMCglmm models. Bar plots are also based on raw data (mean ± SE) to ensure transparency and accurately reflect observed variation. *P* < 0.05 (*), *P* < 0.01 (**), and *P* < 0.001 (***).

Both treatment and time significantly explained the variations in T3 and T4 concentrations (*P* < 0.001, Table 3). As time increased, T3 concentrations exhibited parabolic trends in the PA+ and PA− groups but showed a gradual increase in the Ctrl group (Fig. 3A). Similarly, T4 concentrations showed parabolic trends in the Ctrl and PA− groups, whereas minimal changes were observed in the PA+ group (Fig. 3B). The concentrations of T3 and T4 in the PA−group were significantly higher than those in the PA+ and Ctrl groups during the entire experimental period (*P* = 0.0001, Figs. 3A and 3B, Table 2). The PA− group had the highest concentrations of T3 and T4, followed by the PA+ and Ctrl groups. In summary, as *Eimeria* spp. increased, plateau pikas exhibited lower concentrations of T3 and T4, with the Ctrl group exhibiting the lowest concentrations among the three groups. The interaction between time and treatment had a significant effect on CORT concentration and RMR (*P* = 0.0001, *P* = 0.018, Table 3). With increasing time, the CORT concentration exhibited parabolic trends in the PA+ and PA− groups, whereas an inverted parabolic trend was observed in the Ctrl group (Fig. 3C). Notably, changes in CORT were more pronounced in the mid- and late-stages of the experiment. On D8, the CORT concentration in the PA− group was significantly lower than that in the PA+ and Ctrl groups (*P* = 0.001, *P* = 0.001). On D18, the CORT concentration in the Ctrl group was significantly lower than that in the PA+ and PA− groups (*P* = 0.004, *P* = 0.001, Fig. 3C, Table 2). In terms of RMR, the PA+ group showed inverted parabolic trends over time, whereas the Ctrl and PA− groups showed increasing trends. The changes in RMR were most pronounced in the late stage of the experiment, and on D18, the RMR in the PA+ group was significantly lower than those in the Ctrl and PA− groups (*P* = 0.0001, *P* = 0.002, Fig. 3D, Table 2). Overall, a decreasing trend in the CORT concentration and RMR was observed as *Eimeria* spp. increased.

### Physiological and behavioral correlations

At the among-individual level, we found significant positive correlations between T3 and T4 concentrations (r = 0.564 [0.230, 0.823]), whereas docility was significantly negatively correlated with T3 and T4 concentrations (r = −0.051 [−0.874, −0.026] and r = −0.025 [−0.911, −0.081], respectively). At the within-individual level, RMR was significantly positively correlated with T4 concentration, boldness, and docility (r = 0.424 [0.156, 0.675], r = 0.323 [−0.548, −0.030] and r = 0.330 [0.054, 0.575], respectively), whereas RMR was significantly negatively correlated with exploration (r = −0.314 [0.050, 0.586]). Furthermore, boldness was significantly positively correlated with docility and negatively correlated with CORT concentration (r = 0.351 [0.073, 0.617]). A strong positive within-individual correlation between boldness, RMR, and docility drove the positive phenotypic correlations observed throughout the models, whereas a strong negative within-individual correlation between CORT concentration and boldness drove negative phenotypic correlations (Table 4).

**Table 4:** Results of testing binary correlations between T3, T4, CORT, RMR, exploration, boldness and docility with MCMC general linear mixed effects models. The best estimates of correlation coefficients (values above the diagonal) and their 95% credibility intervals (values below the diagonal) are presented for among-individual, within-individual, and phenotypic correlations in *Ochotona curzoniae*. Significant results corresponding to correlation coefficients whose confidence intervals do not overlap zero are shown in bold.

		T3	T4	CORT	RMR	Exploration	Boldness	Docility
T3	Among-individual	–	**0.564**	0.240	−0.297	−0.601	−0.535	**−0.051**
	Within-individual	–	**0.312**	0.064	0.228	−0.140	0.009	0.237
	Phenotypic	–	**0.470**	0.164	0.010	−0.269	−0.222	0.136
T4	Among-individual	**0.230, 0.823**	–	0.345	−0.370	−0.116	−0.586	**−0.025**
	Within-individual	**0.011, 0.598**	–	0.040	**0.424**	−0.279	0.223	0.213
	Phenotypic	**0.238, 0.697**	–	0.220	0.068	−0.187	−0.151	0.118
CORT	Among-individual	−0.182, 0.628	−0.056, 0.711	–	0.006	0.084	−0.283	−0.008
	Within-individual	−0.254, 0.363	−0.287, 0.355	–	−0.003	0.170	**−0.3003**	0.126
	Phenotypic	−0.134, 0.427	−0.056, 0.495	–	−0.002	0.153	**−0.295**	0.081
RMR	Among-individual	−0.771, 0.289	−0.824, 0.216	−0.634, 0.594	–	0.027	−0.055	−0.152
	Within-individual	−0.066 0.518	**0.156, 0.675**	−0.283, 0.288	–	**−0.314**	**0.323**	**0.330**
	Phenotypic	−0.263, 0.278	−0.216, 0.338	−0.256, 0.249	–	**−0.249**	0.230	**0.259**
Exploration	Among-individual	−0.561, 0.473	−0.542, 0.528	−0.484, 0.444	−0.795, 0.587	–	0.334	−0.123
	Within-individual	−0.080, 0.541	−0.118, 0.512	−0.186, 0.434	**0.050, 0.586**	–	0.011	0.115
	Phenotypic	−0.081, 0.334	−0.088, 0.325	−0.151, 0.290	**0.019, 0.486**	–	0.029	0.093
Boldness	Among-individual	−0.954, 0.061	−0.934, 0.828	−0.758, 0.784	−0.608, 0.649	−0.900, 0.985	–	−0.127
	Within-individual	−0.459, 0.209	−0.601, 0.080	−0.140, 0.438	**−0.548, -0.030**	−0.279, 0.313	–	**0.351**
	Phenotypic	**−0.505, -0.019**	−0.440, 0.070	−0.099, 0.395	−0.468, 0.008	−0.224, 0.273	–	**0.271**
Docility	Among-individual	**−0.874, -0.026**	**−0.911, -0.081**	−0.822, 0.393	−0.607, 0.532	−0.976, 0.915	−0.836, 0.709	–
	Within-individual	−0.302, 0.326	−0.097, 0.522	**−0.562, -0.022**	**0.054, 0.575**	−0.161, 0.419	**0.073, 0.617**	–
	Phenotypic	−0.476, 0.032	−0.418, 0.132	**−0.531, -0.058**	−0.023, 0.475	−0.161, 0.330	**0.038, 0.498**	–

## Discussion

Although parasites are widely recognized as major drivers of host health and behavior, most empirical studies have emphasized their impacts on host health and immune system responses (Johnson & Hoverman, 2012). Recent research has provided new insights into behavioral ecology, revealing the potential effects of parasitic infections on host behavior and personality (Barber & Dingemanse, 2010; Poulin, 2013). *Eimeria* spp., a common endoparasites, can directly affect the physiological status of small mammals by weakening the host immune system, altering energy allocation patterns, and further influencing host personality by changing their responses to predators, foraging, or social behavior (Shivaramaiah et al., 2014). This study assessed the critical role of *Eimeria* spp. infections in driving physiological and personality variations in a native small mammal on the Qinghai-Tibetan Plateau. Our results demonstrated that *Eimeria* spp. parasitism leads to variations in boldness, exploration, docility, RMR, and concentrations of T3, T4, and CORT in plateau pikas and that the interactions between personality and physiology are complex, depending on the parasite infection status.

### Variations in the *Eimeria* spp. numbers

There were significant differences in the *Eimeria* spp. numbers among the three treatments, with the *Eimeria* spp. numbers gradually decreasing over time, while their numbers peaked on D5, consistent with the results of Bian et al. (2011). The proliferation of *Eimeria* spp. within a host typically follows a specific lifecycle. In the PA+ group, *Eimeria* spp. numbers peaked on D5, which was attributed to the maturation of spores as well as the processes of schizogony (asexual reproduction) and parasite proliferation. Following host infection, *Eimeria* spp. rapidly increases in number through a series of developmental processes. The merozoites expanded rapidly through asexual reproduction, reaching a peak in *Eimeria* spp. numbers at approximately D5 (Aguiar-Martins et al., 2024; Bangoura & Daugschies, 2017). Subsequently, *Eimeria* spp. entered the sexual reproduction stage, and their numbers decreased (López-Osorio, Chaparro-Gutiérrez & Gómez-Osorio, 2020; Mesa-Pineda et al., 2021). Because of self-limiting infection, pikas enhance their immune functions to prevent reinfection and restrain parasites, resulting in a rapid decrease in *Eimeria* spp. numbers from D5 to D8 (Garraud et al., 2003). As continuous gavage was not performed throughout the experiment, *Eimeria* spp. numbers gradually decreased owing to the host immune response as the infection progressed (Ghareeb et al., 2022).

Previous studies have demonstrated that increased water intake may facilitate the elimination of internal parasites (Worthington et al., 2013; Zakeri et al., 2018). This is consistent with our finding that there is a gradual decrease in the number of *Eimeria* spp. at the beginning of the saline gavage volume, *i.e*., 5–8 days. This suggests that saline facilitates the excretion of metabolic waste and parasites by diluting intestinal contents (Ahmed, 2023; Halliez & Buret, 2015). However, it is important to clarify that in our study, physiological saline was administered only once at the beginning of the experiment, and no further saline was given afterward. Therefore, the continuous decrease in the number of *Eimeria* spp. in the saline group may be due to changes in the intestinal environment. Saline affects the intestinal pH and electrolyte balance to some extent, thereby altering the intestinal environment, which may help inhibit the growth or reproduction of *Eimeria* spp. (Lee et al., 2025). For example, *Eimeria* infection can lead to a decrease in intestinal pH, which in turn affects the digestion and absorption of nutrients (Bayat et al., 2025). In addition, saline may influence intestinal motility, resulting in accelerated fecal excretion and thereby promoting the expulsion of parasites (Halliez & Buret, 2015). For instance, *Heligmosomoides polygyrus* infection can increase intestinal motility, which facilitates the elimination of parasites (Camberis, Le Gros & Urban, 2003). Additionally, host immune responses play an important role in regulating parasite load, with hosts relying on their immunity to restrain parasitic infections under natural conditions (Lu et al., 2021). As the infection progresses, the immune system of the plateau pikas gradually becomes more effective in controlling and reducing parasite load (Nish & Medzhitov, 2011). Overall, *Eimeria* spp. are regulated by multifactorial interactions rather than isolated parameters. In this study, we reared pikas separately and cleaned their cages daily, which was effective in avoiding repeated *Eimeria* infections. However, *Eimeria* spp. in the Ctrl group were significantly higher than in the other groups on D18, suggesting that the effect of physiological saline was primarily observed during the early stages of infection. It appears to reduce parasite numbers by facilitating excretion, but fails to exert a lasting inhibitory effect on the parasite lifecycle or reproductive capacity (Leggett, Cornwallis & West, 2012; Saracino et al., 2021). Over time, parasites gradually adapt to the host environment and continue to reproduce, with physiological salinity having a limited effect (Leggett, Cornwallis & West, 2012; Saracino et al., 2021). This is consistent with the findings of Orabi et al. (2023), who demonstrated that non-pharmacological interventions are typically effective only in the short term and insufficient to prevent long-term parasite reproduction.

The *Eimeria* spp. in the PA− group were higher than those in the Ctrl group on D5, which may be attributed to individual differences and environmental factors (Cevallos-Gordon et al., 2024). Different individuals metabolize drugs at variable rates, leading to differences in their continued efficacy (Noack, Chapman & Selzer, 2019). Furthermore, the reproductive cycle of coccidia involves multiple stages, including sporulation, invasion of host cells, and schizogony, with the schizogony stage usually lasting several days (Zhou et al., 2019). Consequently, anticoccidial drugs often target specific stages of reproduction, and their efficacy gradually becomes apparent within a few days after administration (Zhang et al., 2023). For example, studies have shown that cedrol has an ACI of 169.34 in chickens, indicating moderate ACI activity, but its significant efficacy usually takes several days to be observed (Zhang et al., 2023). Similarly, in naturally infected lambs, the anticoccidial effect reaches over 90% only 30 days after administration (Pérez-Fonseca, Alcala-Canto & Alberti-Navarro, 2016). These data further suggest that the efficacy of anticoccidial drugs is time-dependent and requires a certain period to manifest. In addition, external environmental factors can also influence the final efficacy of anticoccidial drugs. For example, the density of coccidia in the rodent cage and the nutritional status of the host are key factors (Zdesenko & Mutapi, 2020). As herbivorous animals with coprophagic behavior, pikas contain a high density of coccidia in their feces, which inevitably affects the drug’s efficacy. Although we clean the cages daily, the coprophagic behavior cannot be entirely eradicated (Zdesenko & Mutapi, 2020). The mechanism of anthelmintics typically involves direct inhibition of reproduction or metabolic processes of coccidia, thereby significantly reducing parasite numbers (Lu et al., 2021). However, as the infection cycle progresses, the host gradually develops an adaptive response that diminishes the efficacy of deworming drugs (Flores et al., 2022). Thus, the *Eimeria* spp. was higher in the PA− group than in the PA+ group on D18. These findings suggest that the effectiveness of anticoccidial drugs typically takes some time to fully manifest, which is closely related to the drug’s mechanism of action, pharmacokinetic properties, and the coccidia’s reproductive cycle.

### Effects of *Eimeria* spp. on the personality of plateau pikas

Direct relationships exist between parasite infection intensity and host behavioral variations (Kaushik, Lamberton & Webster, 2012). Because parasites consume energy from their hosts to support their survival and reproduction, they may be more conservative and cautious in their hosts (Li et al., 2024). However, our study found that as the *Eimeria* spp. increased, plateau pikas became more explorative and bold, suggesting that parasites not only depend on the nutrients provided by the host but may also enhance their survival opportunities by altering host metabolism and behavior (Turner et al., 2021). Furthermore, parasitic infections can promote the adaptive evolution of hosts, enhancing their survival by changing their sensitivity and mobility in the environment (Aleuy & Kutz, 2020; Gsell et al., 2023). According to the manipulation hypothesis, parasites can release specific chemicals that affect the nervous system and behavior of the host, making the host more proactive in responding to the environment and benefitting parasite transmission (Pavey & Vyas, 2023; Steffen, 2020).

When *Eimeria* spp. was highest on D5, pikas from the PA+ and PA− groups showed the lowest and highest exploration levels, respectively. At this time, the host is at the peak of infection and the immune system is under maximum stress (Bedford & Apajalahti, 2022). According to the energy cost theory, parasitic infections deplete host energy and increase their metabolic burden, thereby impairing host behavior (Ramnath, 2009). After deworming, the host’s immune system is properly restored, which not only improves health, but also significantly enhances behavioral performance (Bakhtiar et al., 2020; Wammes et al., 2016). On D8, pikas from the Ctrl group showed low *Eimeria* spp. numbers but high exploration, suggesting that hosts can positively adapt to daily activities and maintain stable behavioral performance in the presence of mild parasitic infections (Bruijning et al., 2022). It is noteworthy that parasite number is not the only factor that determines host behavior (Fredensborg, 2014; Gourbière, Morand & Waxman, 2015). Prolonged parasitic infections can overactivate the host immune system, leading to chronic inflammation (Bakhtiar et al., 2020). Anthelmintic drugs effectively eliminate parasites, reduce inflammation, and promote the recovery of the host immune function to baseline levels (Fissiha & Kinde, 2021). Anthelmintic drugs can also help hosts develop a stronger immune tolerance and reduce parasitic infection. This not only protects the host from autoimmune diseases but also improves its resilience to other external stressors (Siddiqui et al., 2023). In addition, strong correlations exist between the host immune system and health. Anthelmintics may help alleviate host physiological symptoms by restoring immune homeostasis and making hosts more proactive and bold (Bennett & Molofsky, 2019; Walsman, Strauss & Hall, 2022). This may explain why *Eimeria* spp. were similar in the PA− and Ctrl groups, but the PA− group showed a higher boldness on D5 and D8.

On D5, when *Eimeria* spp. was the highest in each group, the pikas were more docile, and vice versa. This is consistent with the physiological protective hypothesis, which suggests that hosts exhibit more conservative behavior when facing higher physiological stress (Li et al., 2024). Neurotransmitters are chemicals in the nervous system that transmit signals between the neurons. Important neurotransmitters such as serotonin and dopamine play key roles in regulating behavior and personality. Parasite infections can alter the levels of these neurotransmitters, thereby influencing host behavior (Prandovszky et al., 2011). For example, when serotonin levels are high, crustaceans tend to be more docile but less impulsive and aggressive (Huber et al., 1997). Additionally, a high parasite burden can severely affect the host intestine, causing symptoms such as diarrhea, dehydration, and malnutrition. These physiological changes, in turn, affect host behavior, making them more docile (Ramnath, 2009; Reichert, Bolek & McCullagh, 2023). Future research is needed to explore the relationships between parasite number fluctuations and host behavior variations at different stages of infection to explore the specific mechanisms of parasite-host interactions.

### Effects of *Eimeria* spp. on the physiology of plateau pikas

As expected, *Eimeria* spp. was negatively correlated with T3, T4, and RMR in pikas after infection. This can be attributed to several factors. First, parasite infection can trigger the host immune response and elevate the levels of pro-inflammatory cytokines such as IL-6, TNF-α, and IFN-γ (Maizels & Holland, 1998; Gazzinelli & Denkers, 2006). The production of thyroid hormones is impeded by these cytokines through their interference with the hypothalamic-pituitary-thyroid (HPT) axis and reduction in thyrotropin-releasing hormone (TRH) and thyroid-stimulating hormone (TSH) secretion, thereby suppressing the production of thyroid hormones (Alvarado-Esquivel et al., 2019; Wilkinson & Brown, 2015; Cheng et al., 2023). Studies have shown that chronic inflammation directly suppresses thyroid function, decreases T3 and T4 production, and reduces energy metabolism in the host (Li et al., 2020). Additionally, the metabolic stress and tissue damage induced by *Eimeria* spp. infection, particularly in the intestinal epithelium, significantly affect nutrient absorption, particularly iodine, which is essential for thyroid hormone synthesis (Teng et al., 2021), as well as energy-producing substrates such as carbohydrates and proteins, leading to energy deficiencies (Yuan et al., 2022). Parasite infection often leads to host anorexia and a decrease in body mass, whereas malnutrition and impaired iodine uptake further restrain the host’s thyroid function, resulting in reduced T3 and T4 levels (Ghareeb et al., 2022). Thyroid hormones are essential for the regulation of metabolism, and parasite infections may reduce host metabolism by decreasing the concentration of these hormones, subsequently affecting host energy balance.

As *Eimeria* spp. varied, the PA− group exhibited the highest concentrations of T3 and T4, followed by the PA+ group, whereas the Ctrl group had the lowest concentrations. This suggests that the PA− group reduces parasite burden and decreases the secretion of pro-inflammatory cytokines, thus attenuating the inhibitory effects on the HPT axis and restoring thyroid hormone secretion (Yavropoulou, Sfikakis & Chrousos, 2023). Hormone levels in the PA+ group were higher than those in the Ctrl group, reflecting a compensatory immune response (Nish & Medzhitov, 2011). Thyroid hormone production was significantly suppressed due to the higher *Eimeria* spp. in the PA+ group. In contrast, the Ctrl group showed the lowest long-term negative effects on the endocrine system (Escobedo et al., 2005; King & Li, 2018). All three groups showed a decrease in RMR as the *Eimeria* spp. increased. However, on D5, when *Eimeria* spp. was the highest, the RMR of the PA+ group was significantly greater than that of the Ctrl and PA− groups, whereas the lowest RMR was observed in the PA+ group on D18, when *Eimeria* spp. was the lowest. These results suggest that the energy expenditure of both parasites and hosts aligns with the energy-partitioning model, which posits that animals maintain stable energy balance. According to this model, total energy remains constant, meaning that an increase in energy expenditure in one area leads to a compensatory decrease in the other (Hammond & Diamond, 1997; Montiglio et al., 2018). In the present study, plateau pikas infected with *Eimeria* spp. in the PA+ group consumed additional energy to enhance their immune system, resulting in a lower RMR than those in the PA− and Ctrl groups. This is consistent with the finding that cape ground squirrels infected with both ectoparasites and endoparasites exhibit a lower RMR than squirrels treated with anticoccidial drugs (Scantlebury et al., 2007). We also found significant negative correlations between RMR and exploration, indicating that plateau pikas exhibiting greater exploration tended to have lower metabolic rates than those with lower boldness or exploration abilities. This is consistent with the findings of Careau et al. (2019, 2008), who reported that fall field crickets (*Gryllus pennsylvanicus*) with boldness were characterized by consistently lower standardized metabolic rates, which was interpreted as evidence of an allocation trade-off between activity and maintenance.

Cortisol is a major glucocorticoid hormone produced by the adrenal glands that responds to stress and personality. Parasite infections can stimulate cortisol secretion to reduce attacks on the host immune system and contribute to host fitness (Chao, Alten & Walter, 1994). In this study, we observed a decreasing trend in cortisol levels as the *Eimeria* spp. increased. During the early stages of infection, cortisol levels increase in response to parasitic stress, which can help individuals cope better with parasitic infections (Kotepui et al., 2024). However, in the mid- and late-stages of infection, hosts adapt to parasite stress and cortisol levels recover to normal levels (Alzahrani et al., 2021; Paolo & Nosanchuk, 2006). This adaptive mechanism can help hosts to avoid further damage from parasitism. In addition, parasitic infections may alter the energy metabolism of individuals. In the case of *Eimeria* spp. infection, individuals may allocate more energy to the immune system to defend themselves against infection than to cortisol synthesis (Wensveen, Šestan & Polić, 2024; Otranto & Dantas-Torres, 2013). Therefore, understanding the impact of parasitism on the physiology and behavior of hosts will provide deeper insights into the mechanisms of host-parasite interactions, which can have profound implications for disciplines such as ecology and evolutionary biology. It is important to note that this study was conducted under controlled laboratory conditions. While this setting allowed precise control over parasite load and accurate measurement of host responses, it may also limit the direct extrapolation of our findings to wild populations. The plateau pika (*Ochotona curzoniae*) is a wild species, and its behaviors under laboratory housing conditions (such as exploration, boldness, and docility) may not fully reflect its natural state. The absence of predators, natural social structures, and foraging environments in the laboratory may alter behavioral and physiological responses to parasitic infection. Therefore, while our results provide crucial evidence for the parasite-personality-physiology nexus, future studies conducted in semi-natural or field settings would be valuable to confirm the ecological relevance of these interactions. In future studies, we need to further explore the effects of these treatments on parasite survivability, focus on the effects of parasite viability on host behavior and physiology. Specifically, by integrating fluctuations in parasite numbers across different infection stages and treatments, we aim to estimate parasite survival rates (*i.e*., the ratio of live to dead parasites) after each treatment and clarify how survival dynamics influence host behavioral and physiological responses.

## Conclusions

In conclusion, this work offers empirical support for the effect of parasitism on the behavior and physiology of small mammals in the Qinghai-Tibetan Plateau. Our findings demonstrate that infection with *Eimeria* spp. induces variations in personality, metabolism, and hormone concentrations in plateau pikas. Interactions among behavior, physiology, and parasitic infections are complex. Exploring behavioral and physiological variations, as well as their associations post-infection, is critical for understanding the evolution and adaptation of host-parasite interactions.

## Supplemental Information

10.7717/peerj.20420/supp-1 Supplemental Information 1 The ARRIVE guidelines 2.0: author checklist.

10.7717/peerj.20420/supp-2 Supplemental Information 2 Relative description.

10.7717/peerj.20420/supp-3 Supplemental Information 3 Primary dataset.

## Additional Information and Declarations

### Data Availability

The following information was supplied regarding data availability:

The raw data is available in the Supplemental Files.
